# Acute generalized exanthematous pustulosis following nemolizumab therapy in a patient with atopic dermatitis

**DOI:** 10.1016/j.jdcr.2026.07.036

**Published:** 2026-07-29

**Authors:** David Dunaway, Emma Madigan, Jina Chung, Liam Pettit, Ashely Aniyankunju

**Affiliations:** a Dermatology Partners, Bryn Mawr, Pennsylvania; b University of New England College of Osteopathic Medicine, Portland, Maine; c Department of Dermatology, University of Pennsylvania, Philadelphia, Pennsylvania

**Keywords:** acute generalized exanthematous pustulosis, adverse reaction, AGEP, atopic dermatitis, biologics, drug eruption, nemolizumab

## Introduction

Acute generalized exanthematous pustulosis (AGEP) is a severe adverse reaction that demonstrates the rapid development of sterile, nonfollicular-based pustules on an erythematous base. Onset typically occurs within 24 to 48 hours of administration of the causative medication and presents with fever, rash, and leukocytosis.^1^ A wide range of medications have been reported to cause AGEP, with antibacterials being the most common.^2^ AGEP is a rare condition, with an estimated annual incidence of 1 to 5 million people worldwide.^3^ Nemolizumab is a humanized monoclonal antibody targeting the alpha subunit of the interleukin (IL) 31 receptor.^4^ IL-31 is a neuroimmune cytokine that plays a role in the perpetuation of itch and the primary pathogenesis of atopic dermatitis, with the IL-31 receptor being expressed by keratinocytes, immune cells, and neurons.^4^ The most commonly reported cutaneous adverse events following nemolizumab are eczema or exacerbation of atopic dermatitis, erythema, neurodermatitis, acne, and urticaria.^5^ In this report, we describe a case of AGEP following nemolizumab treatment in a 55-year-old Caucasian woman.

## Case report

A 55-year-old woman presented to our clinic with inadequately controlled atopic dermatitis. Failed treatments include topical corticosteroids, topical calcineurin inhibitors, and topical ruxolitinib with minimal benefit. She also had been treated with dupilumab for approximately 1 year but developed 2 episodes of conjunctivitis. So, dupilumab was discontinued. After discussing the remaining advanced therapy options, the decision was made to pursue treatment with nemolizumab.

On day 0, a loading dose of 60 mg of nemolizumab was administered subcutaneously in the office. On day 28, the patient reported worsening of her eczema, which was attributed to the flaring-remitting nature of atopic dermatitis, and the decision to continue nemolizumab was made. On days 28 and 57, she received her second and third injections of 30 mg of nemolizumab, respectively. On day 58, the patient reported developing a diffuse rash on her neck, behind her ears, and on the flexural surface of her extremities. The patient described the rash as an “itching and burning feeling” and noted drainage from the rash near her ears. The patient presented to an urgent care clinic, where she was prescribed a 60-mg taper dose of prednisone. Urgent care did not order any laboratory testing. She affirmed temporary improvement in itching with the oral steroid but denied clearance of the rash. She denied fevers and chills. She denied starting any new medications or taking any antibiotics or antifungals. Her list of active medications included fluticasone/salmeterol inhaler, estradiol/norethindrone tablet, and tirzepatide injection, all of which had been started more than 3 months prior to nemolizumab.

Thirty-three days after starting prednisone taper, she presented to our clinic. On examination, we observed scaly erythematous patches on her trunk and proximal extremities with a predilection for the postauricular scalp, side of the neck, and flexural surface of her upper and lower extremities (Fig 1). Scattered throughout these patches were small, nonfollicular-based pustules. At this visit, she provided photos that revealed many studded, coalescing, nonfollicular-based pustules along the ear, neck, and arms. With the morphology being concerning for AGEP, a punch biopsy was performed of a remaining pustule on her back. A complete metabolic panel and complete blood count were also ordered to rule out drug rash with eosinophilia and systemic symptoms, although there was low clinical suspicion given the lack of facial erythema and edema. The patient was instructed to discontinue nemolizumab and start topical triamcinolone 0.1% cream twice daily.

**Fig 1:**
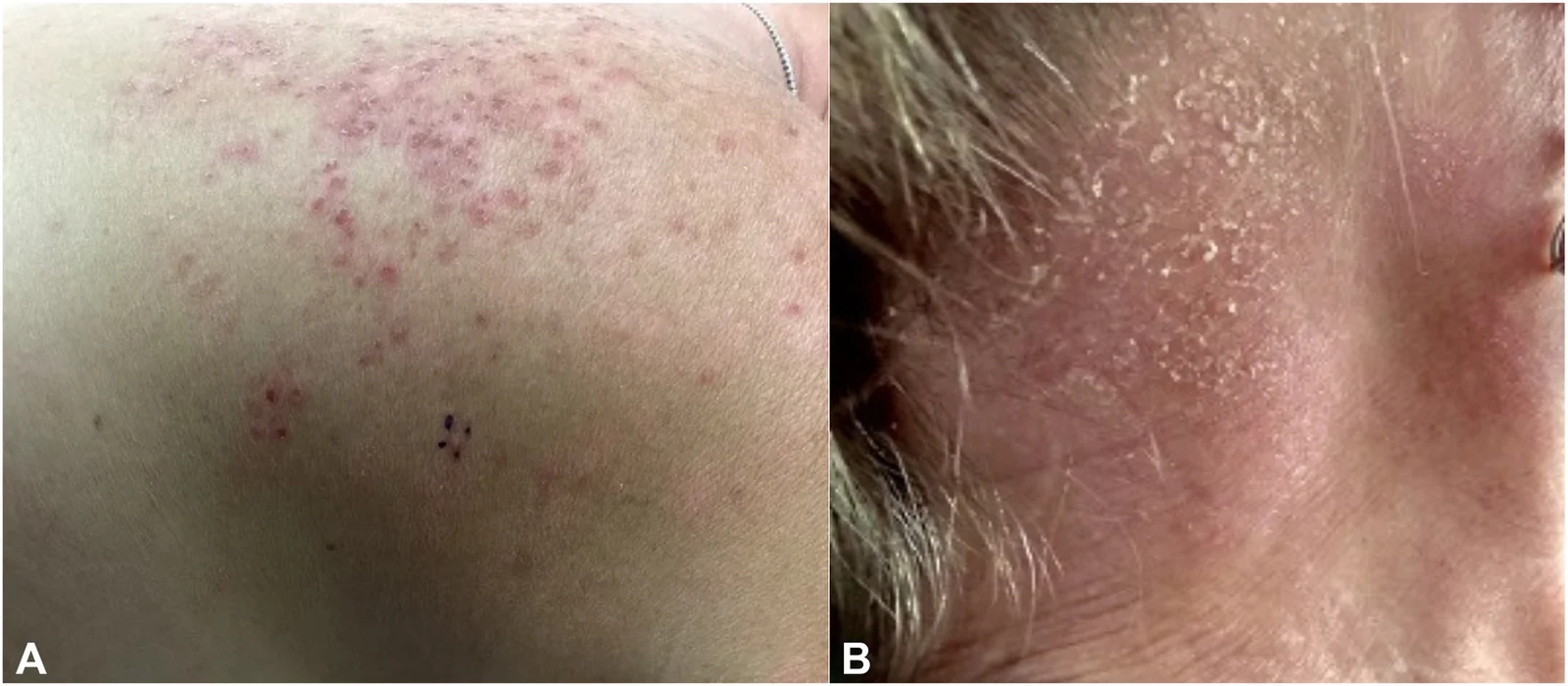
**A,** Scaly, erythematous patches with scattered, nonfollicular-based pustules on the trunk, with a purple skin marking pen denoting the biopsy site. **B,** Scaly, erythematous patches with few pustules on the postauricular neck.

Histopathologic examination of the specimen showed subcorneal pustules with neutrophils and a superficial lymphocytic infiltrate with numerous neutrophils and eosinophils, consistent with the clinical diagnosis of AGEP (Fig 2). The EuroSCAR study group scoring system score was 5, signifying a probable diagnosis of AGEP (Table I).^2^ The patient followed up in the clinic 2 weeks later, 2 months after her last administration of nemolizumab, with only slight improvement in her rash and continued itch. On physical examination, the patient had persistent erythematous patches with scale but no new pustules. The patient was advised of the long half-life of nemolizumab and educated that it could take approximately 4.5 half-lives for the medication to be eliminated from her system. Given her persistent severe pruritus and eczematous patches, upadacitinib 15 mg daily was initiated to manage both her underlying atopic dermatitis and her ongoing AGEP, with eventual complete resolution.

**Fig 2:**
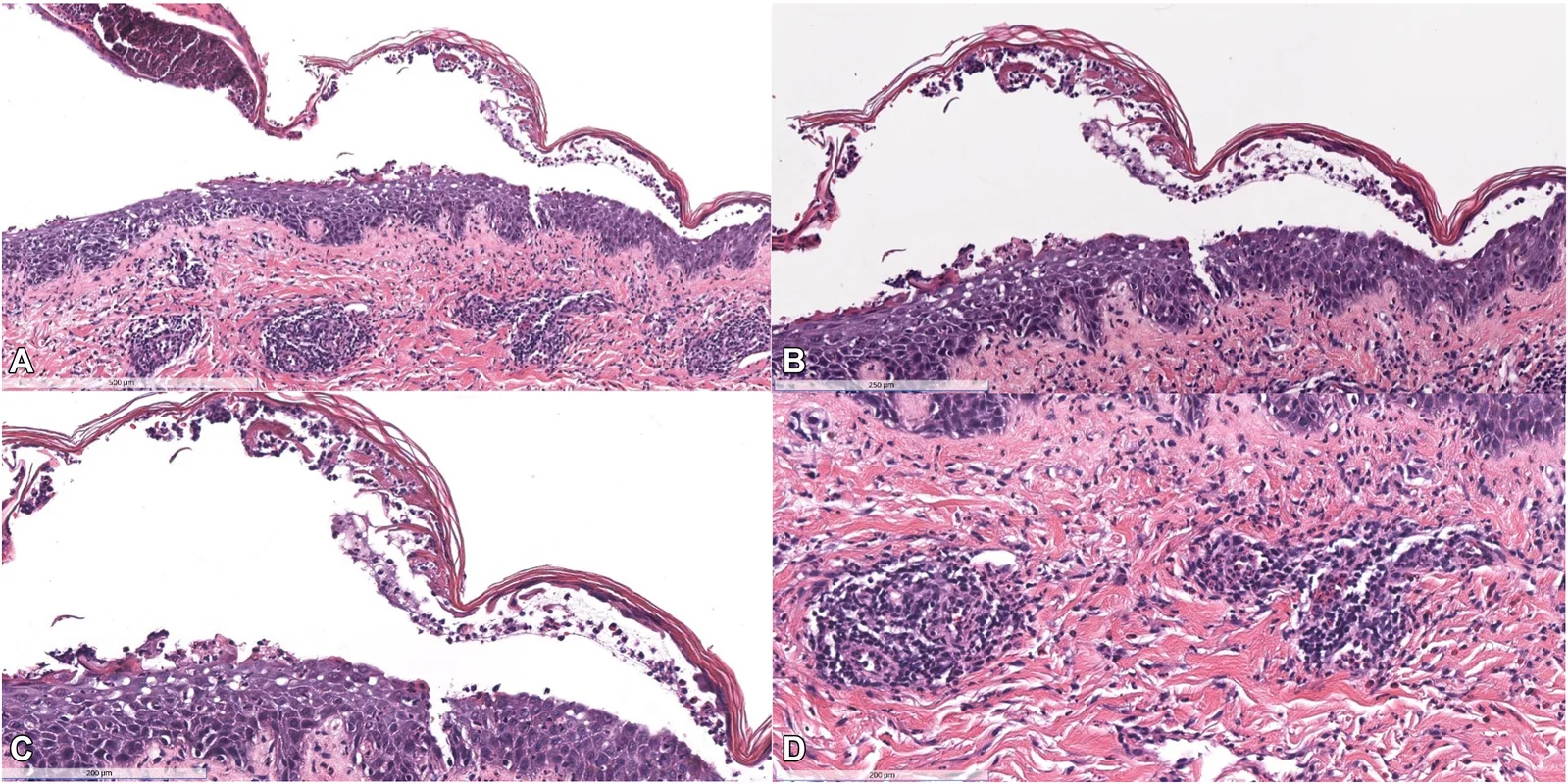
**A,** Subcorneal pustules with neutrophils and a superficial lymphocytic infiltrate with numerous neutrophils and eosinophils at lower magnification. **B, C,** Higher magnification views demonstrating neutrophils in subcorneal pustules. **D,** Superficial lymphocytic infiltrate with neutrophils and eosinophils.

**Table I:** EuroSCAR Study Group Scoring System

Criteria	Description	Score
Morphology		
Pustules	Typical, sterile, nonfollicular	+2
Erythema	Typical, diffuse	+2
Distribution/pattern	Face, neck, flexural regions of upper and lower extremities	+2
Postpustular desquamation	Yes (especially demonstrated in Fig 1, *B*)	+1
Course		
Mucosal involvement	No	0
Acute onset	Yes (within 24 h)	0
Resolution of pustules and erythema ≤15 d	No	–4
Fever (≥38 °C)	No	0
Blood neutrophil count (≥7000 cells/mm^3^)	No	0
Histopathologic analysis		
Skin lesions	Subcorneal pustules and superficial lymphocytic infiltrate	+2
Overall score		5

## Discussion

Multiple cutaneous adverse effects were reported during clinical trials for nemolizumab, with the most common being eczema.^5^ At the time of writing of this case, according to our review of preexisting literature, only one other case of AGEP has been reported in association with nemolizumab.^6^

This patient’s presentation is uncommon given the length of presentation of her rash. The half-life of nemolizumab is reported to be 16.7 ± 4.1 days.^7^ This pharmacokinetic profile correlates with the clinical onset and resolution of this patient’s rash, with the patient reporting skin clearance after approximately 90 days, which correlates with the 4.5 half-life timeframe (Fig 3). A limitation in this case is that our patient took oral corticosteroids for approximately 1 month prior to presenting to our clinic. This may have contributed to the relatively mild cutaneous presentation seen on examination compared to typical cases of AGEP.

**Fig 3:**
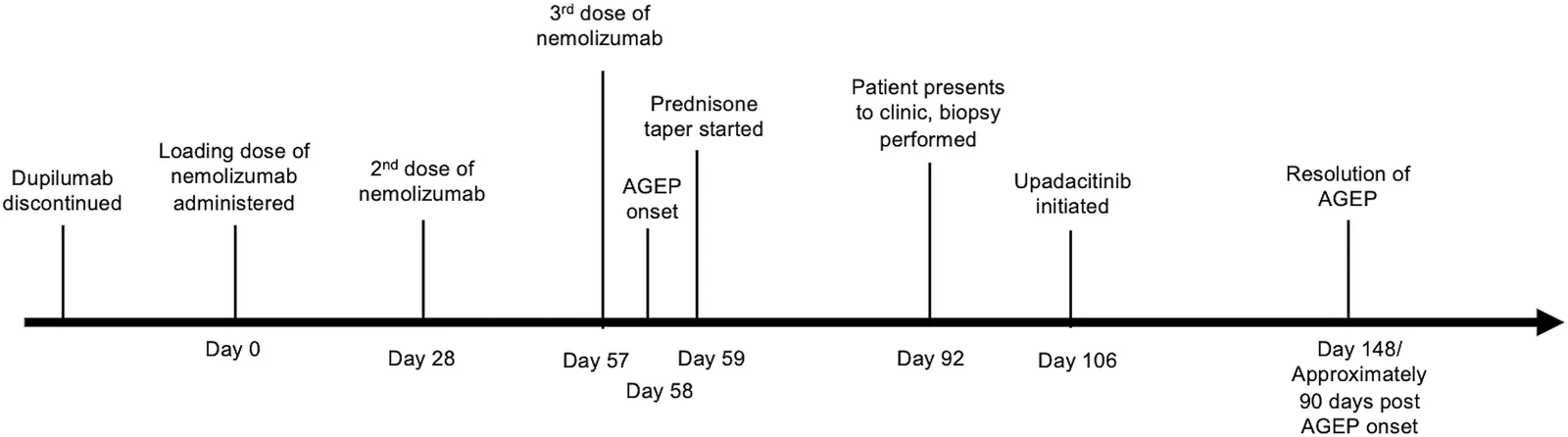
Timeline of this patient’s clinical course, from initiation of nemolizumab to resolution of acute generalized exanthematous pustulosis.

Although the pathogenesis of nemolizumab-induced AGEP remains unclear, IL-31 blockade may disrupt immune homeostasis and promote a shift toward T-cell helper 17/IL-23–mediated inflammation.^8^ A key downstream effect of this is IL-17–driven induction of keratinocytes to produce IL-8, a potent neutrophil chemoattractant.^9^ IL-17 also directly induces IL-36γ expression in keratinocytes, leading to further production of IL-8, chemokine (C-X-C motif) ligand 1, and additional IL-36γ.^10^ Although speculative, this pathway provides a plausible mechanism for the neutrophilic epidermal inflammation observed in AGEP, although a direct link to IL-31 inhibition has not yet been established.

In the modern era of medicine, with an increasing number of novel therapies emerging in the field, it is important to consider uncommon reactions in the differential diagnosis for patients on these medications. This case demonstrates the importance of continued pharmacologic vigilance and the consideration of a broad differential diagnosis when a patient on a systemic medication presents with an adverse reaction, regardless of whether it has been previously reported.

## Conflicts of interest

Dr Dunaway serves as a nonbranded speaker for AbbVie. Authors Madigan, Pettit, and Aniyankunju and Dr Chung and Dr Liam have no conflicts of interest to declare.
